# Supplementary material for: Enhanced exercise and regenerative capacity in a mouse model that violates size constraints of oxidative muscle fibres
Source: eLife. 2016 Aug 5;5:e16940. doi: 10.7554/eLife.16940 (PMC4975572; doi:10.7554/eLife.16940)
Supplement: Supplementary file 1. — DOI: http://dx.doi.org/10.7554/eLife.16940.015 [file elife-16940-supp1.docx]

**Supplementary information**

**Primary antibodies**

| Antigen | Species | Dilution | Supplier |
| --- | --- | --- | --- |
| MYHCI | Mouse | 1:1 | DSHB A4.840 |
| MYHCIIA | Mouse | 1:1 | DSHB A4.74 |
| MYHCIIB | Mouse | 1:1 | DSHB BF.F3 |
| Pax7 | Mouse | 1:1 | DSHB |
| MyoD | Rabbit | 1:200 | Santa Cruz Biot # sc-760 |
| Laminin | Rabbit | 1:200 | Sigma L9393 |
| CD31 | Rat | 1:40 | AbD serotec MCA2388 |
| F4/80 | Rat | 1:100 | Bio-RAD MCA4978 |
| MYH3 | Mouse | 1:200 | Santa Cruz Bio, sc-53091 |
| Cleaved Caspase-3 | Rabbit | 1:200 | Cell signalling Technology #9664S |
| Dystrophin | Rabbit | 1:200 | Abcam 15277 |
| AKT | Rabbit | 1:200 | Cell signalling Technology #4060 |
| pAkt (Ser473) | Rabbit | 1:200 | Cell signalling Technology #9272 |
| 4E-BP1 (53H11) | Rabbit | 1:200 | Cell signalling Technology #9644 |
| p4EBP1 (T37/46) | Rabbit | 1:200 | Cell signalling Technology #9459 |
| FoxO3a (H-144) | Rabbit | 1:200 | Cell signalling Technology #9466 |
| pFoxO3a (Ser253) | Rabbit | 1:200 | Cell signalling Technology SC-11351 |

**Secondary antibodies:**

| Antibody | Species | Dilution | supplier |
| --- | --- | --- | --- |
| Alexa fluor 633 anti-mouse | Goat | 1:200 | Life Technologies # A20146 |
| Alexa fluor 488 anti-mouse | Goat | 1:200 | Life Technologies # A11029 |
| Alexa fluor 488 anti-rabbit | Goat | 1:200 | Life Technologies # A11034 |
| Alexa fluor 594 anti-rabbit | Goat | 1:200 | Life Technologies # A11037 |

**qPCR primers Sequence**

|  | |
| --- | --- |
| **Oligo Name** | **Sequence** |
| R_mERRg.F | ACT TGG CTG ACC GAG AGT TG |
| R_mERRg.R | GCC AGG GAC AGT GTG GAG AA |
| R.mPGC1A.F | AAC CAC ACC CAC AGG ATC AGA |
| R.mPGC1A.R | TCT TCG CTT TAT TGC TCC ATG A |
| R_mPERM1.F | CCTGGTCGTAAGAAGAGGCG |
| R_mPERM1.R | CTTGGGCCTGGTAAGCTGT |
| R_mGlut1.F | CTCTGTCGGCCTCTTTGTTAAT |
| R_mGlut1.R | CCAGTTTGGAGAAGCCCATAAG |
| R_mGlut4.F | ACACTGGTCCTAGCTGTATTCT |
| R_mGlut4.R | CCAGCCACGTTGCATTGTA |
| R_mPDK4.F | AAG CAA AAC ACA AAC ACG AGT A |
| R_mPDK4.R | CCC GGG TCA TCC AAC CA |
| R_mHAD.F | GCTGGGCCTAACTTTGAGTATG |
| R_mHAD.R | CAAAATCAGCGTCATCAGGAGAA |
| R_mLPL.F | GCTGGGCCTAACTTTGAGTATG |
| R_mLPL.R | CAAAATCAGCGTCATCAGGAGAA |
| R_mcycs.F | CCA AAT CTC CAC GGT CTG TTC |
| R_mcycs.R | ATC AGG GTA TCC TCT CCC CAG |
| R_mCat.F | GGATTATGGCCTCCGAGATCTT |
| R_mCat.R | TAAAACGTCCAGGACGGGTAA |
| R_mMYOG.F | CTG TTT AAG ACT CAC CCT GAG AC |
| R_mMYOG.R | GGT GCA ACC ATG CTT CTT CA |
| R_mCD36.F | AGATGACGTGGCAAAGAACAG |
| R_mCD36.R | CCTTGGCTAGATAACGAACTCTG |
| R_mSlc25a20.F | CAACCACCAAGTTTGTCTGGA |
| R_mSlc25a20.R | CCCTCTCTCATAAGAGTCTTCCG |
| R_mFATP1.F | AGGTCAATGAGGACACGATGGAG |
| R_mFATP1.R | CTGGTACATTGAGTTAGGGTCCAAC |
| R_mFabp3.F | ACCTGGAAGCTAGTGGACAG |
| R_mFabp3.R | TGATGGTAGTAGGCTTGGTCAT |
| R_mACADL.F | TGCCCTATATTGCGAATTACGG |
| R_mACADL.R | CTATGGCACCGATACACTTGC |
| R_mACADM.F | CCAGAGAGGAGATTATCCCCG |
| R_mACADM.R | TACACCCATACGCCAACTCTT |
| R_mVEGFA165.F | TGC AGG CTG CTG TAA CGA TG |
| R_mMVEGFA165.R | GAA CAA GGC TCA CAG TGA TTT TCT |
| R_mVEGFA189.F | TGC AGG CTG CTG TAA CGA TG |
| R_mVEGFA189.R | CTC CAG GAT TTA AAC CGG GAT T |
| R_mFGF1.F | GAAGCATGCGGAGAAGAACTG |
| R_mFGF1.R | CGAGGACCGCGCTTACAG |
